# Comparison of techniques for left subclavian artery preservation during thoracic endovascular aortic repair: A systematic review and single-arm meta-analysis of both endovascular and surgical revascularization

**DOI:** 10.3389/fcvm.2022.991937

**Published:** 2022-09-15

**Authors:** Yuchong Zhang, Xinsheng Xie, Ye Yuan, Chengkai Hu, Enci Wang, Yufei Zhao, Peng Lin, Zheyun Li, Fandi Mo, Weiguo Fu, Lixin Wang

**Affiliations:** ^1^Department of Vascular Surgery, Zhongshan Hospital, Fudan University, Xiamen, China; ^2^Department of Vascular Surgery, Zhongshan Hospital, Fudan University, Shanghai, China; ^3^Vascular Surgery Institute of Fudan University, Shanghai, China; ^4^National Clinical Research Center for Interventional Medicine, Shanghai, China; ^5^Fudan Zhangjiang Institute, Shanghai, China

**Keywords:** meta-analysis, left subclavian artery, thoracic endovascular aortic repair, revascularization, endoleak

## Abstract

**Background:**

Currently, the optimal technique to revascularize the left subclavian artery (LSA) during thoracic endovascular aortic repair (TEVAR) remains controversial. Our study seeks to characterize early and late clinical results and to assess the advantages and disadvantages of endovascular vs. surgical strategies for the preservation of LSA.

**Methods:**

PubMed, Embase and Cochrane Library searches were conducted under the PRISMA (Preferred Reporting Items for Systematic review and Meta-Analyses) standards. Only literature published after January 1994 was included. Studies reporting on endovascular revascularization (ER), surgical revascularization (SR) for LSA preservation were included. 30-day mortality and morbidity rates, restenosis rates, and rates of early and late reintervention are measured as outcomes.

**Results:**

A total of 28 studies involving 2,759 patients were reviewed. All articles were retrospective in design. Single-arm analysis found no significant statistical differences in ER vs. SR in terms of 30-day mortality and perioperative complication rates. The mean follow-up time for the ER cohort was 12.9 months and for the SR cohort was 26.6 months, respectively. Subgroup analysis revealed a higher risk of perioperative stroke (4.2%) and endoleaks (14.2%) with the chimney technique compared to the fenestrated and single-branched stent approaches. Analysis of the double-arm studies did not yield statistically significant results.

**Conclusion:**

Both ER and SR are safe and feasible in the preservation of LSA while achieving an adequate proximal landing zone. Among ER strategies, the chimney technique may presents a greater risk of neurological complications and endoleaks, while the single-branched stent grafts demonstrate the lowest complication rate, and the fenestration method for revascularization lies in an intermediate position. Given that the data quality of the included studies were relatively not satisfactory, more randomized controlled trials (RCTs) are needed to provide convincing evidence for optimal approaches to LSA revascularization in the future.

## Introduction

Thoracic endovascular aortic repair (TEVAR) has attracted much attention as a procedure for treating most descending aortic pathologies (1). Standard TEVAR recommends a proximal anchoring zone of no less than 20 mm (2). In many cases, stents will need to be released proximally to cover the left subclavian artery (LSA) in order to expand the landing zone (3). However, direct coverage of LSA during TEVAR may pose a higher incidence of various complications, leading to devastating outcomes including stroke, spinal cord ischemia, or left upper extremity ischemia (4, 5).

In recent years, more and more studies have shown that performing revascularization of the LSA rather than direct coverage may lead to a more favorable overall prognostic outcome (6, 7). Prophylactic LSA revascularization allowing better preservation of normal perfusion through vital branches and thus reducing the risk of potential complications (8). There is a general consensus that revascularization of the LSA should always be performed in most of the case, especially elective surgery (9).

Traditional revascularization usually takes the form of surgical revascularization (SR) such as graft bypass or transposition (10). Endovascular revascularization (ER) techniques such as chimney grafts, fenestrated stent-grafts and single-branched stent-grafts, have developed as an alternative approach over the past two decades (11–13). Open surgery can provide definite results, but may theoretically result in more trauma; whereas, the endovascular technique is currently undergoing different stages of trials and its outcomes are yet to be confirmed.

Controversy still existed regarding the optimal choice of revascularization to minimize the incidence of perioperative complications during TEVAR (12). The objective of the present study was to carry out a meta-analysis of all published studies in an effort to obtain quantitative insight into the impact of various revascularization techniques on the outcome of TEVAR.

## Materials and methods

### Search strategy

The Preferred Reporting Items for Systematic Reviews and Meta-Analyses (PRISMA) Statement was used to guide the conduct of this study (14). We performed a comprehensive search on the following databases: PubMed, Embase, the Cochrane Library. We have no restrictions on the type of publications. With regard to the year of publication, we restricted the period to papers published after January 1994. The search was updated to June 1, 2021. The primary search terms were (i) “revascularization”; (ii) “TEVAR” OR “TEVAR”; and (iii) MeSH term: “Subclavian Artery” which was found in the MeSH hierarchy. Both the “AND” and “OR” operators combined with distinct search terms were used to ensure the integrity of the search results. Relevant articles were also checked in the reference list.

### Inclusion and exclusion criteria

In order to select studies for inclusion in this review, the following criteria were considered: (i) language in English; (ii) more than 2 patients; (iii) publication between January 1994 and August 2021 with adequate data on postprocedural complications and outcomes; (iv) both prospective and retrospective studies. Exclusion criteria were as follows: (i) studies that did not involve humans; (ii) publications categorized as abstracts, letters, editorials, experts’ opinions, reviews, case reports, or technical notes; guidelines; and technical notes; (iii) studies lacking sufficient data for analysis; (iv) duplicate articles or identical data.

### Data extraction

Each candidate study was reviewed by the investigators and the data was then extracted. We retrieved full-text versions of articles that were unable to be categorized by title and abstract alone.

The following items were recorded for each study: Early outcomes including 30-day mortality; stroke rates; spinal cord ischemia rates; endoleak rates; left limb ischemia rates and early reintervention rates. Mid-term or long-term outcomes: follow-up length; cumulative patency rates; late reintervention rates; restenosis or re-occlusion rates.

The definitions of measurements and treatments associated with TEVAR are strictly consistent with *reporting standards for thoracic endovascular aortic repair (TEVAR)* (15). Comprehensive analysis focused primarily on perioperative mortality, stroke, spinal cord ischemia, and endoleak. The three above-mentioned authors completed a quality assessment based on GRADE—Grading of Recommendations, Assessments, Development and Evaluations (16).

### Statistical analysis

Data analysis was conducted following the guidelines outlined in the *Cochrane Handbook for Systematic Reviews of Interventions* (17). The comparative studies (ER vs. SR) were pooled as risk differences (RD) with 95% confidence intervals (CI) and analyzed in a meta-analysis. Single-arm studies were analyzed in a pooled proportion meta-analysis, these data and single proportions were calculated as overall proportions with 95% CI in order to summarize postoperative data and outcomes in each study. The R package Meta were implemented to conduct all analyses (18). Every single proportions were transformated by Freeman-Tukey Double arcsine transformation and passed the normality test before final calculations. A combination of fixed- and random-effects models were used for sensitivity analyses, as well as to calculate the pooled RD and 95% CIs. A subgroup analysis has been carried out in order to examine and explain the diversity of results (heterogeneity) among the various studies. The *I*^2^ statistic was used to assess heterogeneity across the studies. For case of high heterogeneity (*I*^2^ > 50%), a random-effects model was applied, while in case of low heterogeneity (*I*^2^ < 50%), a fixed-effect model was applied. Sensitivity analyses were performed by comparing the output of both fixed- and random-effects models. Visual analyses of funnel plots and Egger regression tests were performed to evaluate publication bias. Statistical significance was defined as a *p*-value of < 0.05 with two-sided CI. All statistical analyses were carried out using R software (version 4.1.3).^1^

## Results

### Data from overall studies

#### Search results and study selection

**FIGURE 1:**
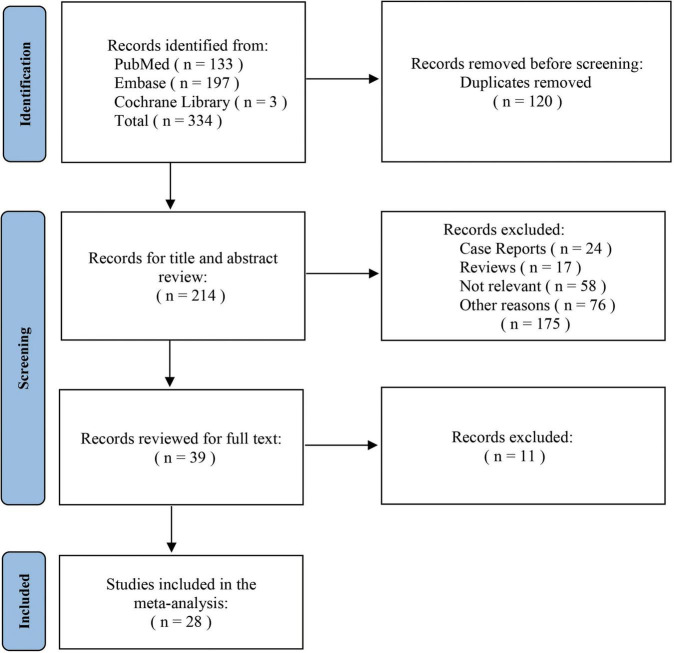
Flow chart illustrating the process of selecting studies.

The initial search identified 334 studies associated with the topic. Of these, 214 publications remained after calculating duplications. After screening titles and abstracts, 24 case reports, 17 reviews and 134 publications that did not meet the inclusion criteria were excluded. All remaining 39 studies were retrieved for full text, of which 28 articles were finally included for the analysis (19–46). The screening procedure for this review is depicted in Figure 1. The meta-analysis included a total of 2,759 patients. ER data were derived from 12 studies involving 360 patients to 22 studies involving 2,399 patients for the SR cohort. The number of cases included in cohorts implementing chimney method, fenestration technique and single-branched stent were 72, 194, and 94, respectively.

#### Early outcomes

Cumulative 30-day mortality was 0.4% (95% CI: 0.0–1.9%) for ER and 2.8% (95% CI: 1.3–4.9%) for SR, respectively. The incidence of early stroke was 0.1% (95% CI: 0.0–1.2%) for ER and 4.1% (95% CI: 2.6–5.8%) for SR. In ER cohort, spinal cord ischemia was observed in 0.0% (95% CI: 0.0–0.8%) of the cases, while in SR, the incidence was 1.7% (95% CI: 1.1–2.4%). Left arm claudication was recorded respectively, in ER and SR with 0.0% (95% CI: 0.0–0.3%) and 1.7% (95% CI: 0.4–3.4%). As for Endoleak, the incidence in ER is 8.3% (95% CI: 2.1-17.1%) and the incidence in SR is 11.2% (95% CI: 4.4–20.5%; Table 1). The overall proportions of early reinterventions were 2.0% (95% CI: 0.0–17.0%) for ER and 9.4% (95% CI: 7.3–11.7%; Table 2) for SR.

**TABLE 1:** Data on early and late outcomes following endovascular revascularization and surgical revascularization.

Author/study/year	30-day mortality *n* (%)	Stroke *n* (%)	Spinal cord ischemia *n* (%)	Left arm claudication *n* (%)	Endoleak *n* (%)	Restenosis *n* (%)
**Endovascular**						
**Chimney**						
Piffaretti et al. (19)	2 (6.4)	1 (3.2)	0 (0)	0 (0)	4 (12.9)	NR
Xiang et al. (20)	0 (0)	0 (0)	1 (4.1)	NR	10 (41.6)	NR
Ramdon et al. (21)	0 (0)	3 (17.6)	0 (0)	0 (0)	0 (0)	NR
Cumulative data	1.6% 95%CI:0.0–6.9%	4.2% 95%CI: 0.0–16.8%	1.8% 95%CI: 0.0–7.2%	0.0% 95%CI:0.0–3.9%	14.2% 95%CI: 0.0–44.6%	/ /
**Fenestration**						
Ahanchi et al. (22)	0 (0)	0 (0)	0 (0)	NR	0 (0)	NR
Redlinger et al. (23)	1 (4.5)	0 (0)	0 (0)	0 (0)	2 (9.1)	1 (4.5)
Bradshaw et al. (24)	NR	1 (3)	0 (0)	NR	NR	NR
Qin et al. (25)	1 (4.1)	0 (0)	0 (0)	NR	0 (0)	NR
Wang et al. (26)	0 (0)	0 (0)	NR	NR	1 (5.8)	0 (0)
Luo et al. (27)	0 (0)	0 (0)	0 (0)	0 (0)	13 (26.0)	NR
Xie et al. (46)	0 (0)	0 (0)	1 (2.3)	0 (0)	NR	NR
Cumulative data	0.1% 95%CI:0.0–2.1%	0.0% 95%CI: 0.0–1.1%	0.0% 95%CI: 0.0–1.2%	0.0% 95%CI:0.0–0.8%	10.2% 95%CI: 4.7–17.0%	1.4% 95%CI:0.0–9.4%
**Single-branched**						
Huang et al. (28)	0 (0)	0 (0)	NR	0 (0)	1 (4.7)	NR
Fang et al. (29)	1 (1.3)	0 (0)	0 (0)	0 (0)	3 (4.1)	NR
Cumulative data	0.6% 95%CI: 0.0–4.1%	0.0% 95%CI: 0.0–1.6%	0.0% 95%CI: 0.0–1.7%	0.0% 95%CI: 0.0–1.6%	3.9% 95%CI: 0.5–9.3%	/
Cumulative data	0.4% 95%CI: 0.0–1.9%	0.1% 95%CI:0.0–1.2%	0.0% 95%CI:0.0–0.8%	0.0% 95%CI: 0.0–0.3%	8.3% 95%CI: 2.1–17.1%	1.7% 95%CI: 0.0–9.9%
**Surgery**						
Iida et al. (30)	0 (0)	2 (10.5)	1 (5.2)	0 (0)	0 (0)	NR
Lee et al. (31)	2 (6.2)	1 (3.1)	1 (3.1)	NR	1 (3.1)	NR
Madenci et al. (32)	6 (6.8)	5 (5.7)	NR	NR	NR	NR
Scali et al. (33)	7 (6.9)	9 (8.9)	6 (5.9)	NR	NR	NR
Contrella et al. (34)	4 (9)	6 (14)	0 (0)	2 (5)	NR	NR
Saouti et al. (35)	NR	0 (0)	0 (0)	5 (9.8)	NR	1 (2.0)
Zamor et al. (36)	0 (0)	2 (3.3)	2 (3.3)	0 (0)	NR	NR
Bradshaw et al. (24)	NR	0 (0)	0 (0)	NR	NR	NR
Canaud et al. (37)	7 (12.7)	0 (0)	2 (3.6)	NR	NR	NR
Kamman et al. (38)	0 (0)	8 (6.9)	1 (1.4)	1 (1.4)	26 (35.1)	NR
Piffaretti et al. (19)	1 (2.4)	1 (2.4)	1 (2.4)	0 (0)	1 (2.4)	NR
van der Weijde et al. (39)	0 (0)	2 (1.9)	3 (2.9)	2 (1.9)	NR	NR
Xiang et al. (20)	1 (7.1)	0 (0)	1 (7.1)	NR	4 (28.6)	NR
Delafontaine et al. (40)	51 (8.7)	56 (9.6)	7 (1.2)	47 (8.1)	NR	NR
Bartos et al. (41)	2 (2.9)	3 (4.3)	2 (2.9)	2 (2.9)	NR	NR
Bianco et al. (42)	3 (5.2)	2 (3.4)	4 (6.9)	NR	7 (12.1)	NR
D’Oria et al. (43)	24 (3)	33 (4.6)	25 (3.5)	10 (1.4)	NR	NR
Ramdon et al. (21)	2 (3.1)	5 (7.8)	0 (0)	0 (0)	3 (4.6)	NR
Wang et al. (26)	0 (0)	1 (3.1)	NR	NR	3 (9.4)	0 (0)
Johnson et al. (44)	0 (0)	1 (2.9)	1 (2.9)	0 (0)	9 (25.7)	NR
Parker et al. (45)	3 (8.6)	1 (2.9)	NR	NR	NR	NR
Xie et al. (46)	0 (0)	2 (2.0)	1 (1.0)	2 (2.0)	NR	NR
Cumulative data	2.8% 95%CI: 1.3–4.9%	4.1% 95%CI:2.6–5.8%	1.7% 95%CI: 1.1–2.4%	1.7% 95%CI: 0.4–3.4%	11.2% 95%CI: 4.4–20.5%	0.9% 95%CI: 0.0–4.9%

CI, Confidence interval; NR, not reported.

**TABLE 2:** Follow-up times, reinterventions and patency rates after endovascular revascularization and surgical revascularization.

Author/study/year	Mean follow-up (months)	Early reinterventions *n* (%)	Late reinterventions *n* (%)	Cumulative patency (%)	Quality assessment
**Endovascular**					
**Chimney**					
Piffaretti et al. (19)	24 ± 21	NR	2 (6.4)	100	Moderate
Xiang et al. (20)	21.3 ± 10.8	NR	3 (12.5)	95.8	Moderate
Ramdon et al. (21)	NR	NR	NR	100	Moderate
Cumulative data	22.8 ± 16.5	/	8.6% 95%CI: 2.0–18.2%	99.4% 95%CI: 95.0–100.0%	
**Fenestration**					
Ahanchi et al. (22)	8	NR	NR	100	Low
Redlinger et al. (23)	11	2 (9.1)	2 (9.1)	95.4	Low
Bradshaw et al. (24)	24	0 (0)	NR	NR	Moderate
Qin et al. (25)	10	NR	NR	100	Moderate
Wang et al. (26)	12.7 ± 9.3	NR	NR	NR	Low
Luo et al. (27)	15	NR	2 (4)	100	Low
Xie et al. (46)	NR	NR	NR	NR	Low
Cumulative data	15.1 ± 1.0	1.6% 95%CI: 0.0–16.6%	3.9% 95%CI: 0.1–10.9%	100.0% 95%CI: 97.6–100.0%	
**Single-branched**					
Huang et al. (28)	12	NR	NR	100	Low
Fang et al. (29)	1	NR	NR	NR	Moderate
Cumulative data	3.5	/	/	99.9% 95%CI:91.4–100.0%	
Cumulative data	12.9 ± 3.6	2.0% 95%CI: 0.0–17.0%	5.9% 95%CI: 1.9–11.4%	99.9% 95%CI: 98.1–100.0%	
**Surgery**					
Iida et al. (30)	27	NR	NR	NR	Very low
Lee et al. (31)	NR	NR	NR	NR	Low
Madenci et al. (32)	NR	NR	NR	NR	Moderate
Scali et al. (33)	12.0 ± 19.4	NR	6 (5.9)	94	Moderate
Contrella et al. (34)	27.5	NR	NR	NR	Low
Saouti et al. (35)	27.6	NR	NR	NR	Very low
Zamor et al. (36)	24.9	NR	NR	100	Moderate
Bradshaw et al. (24)	24	3 (14.3)	NR	NR	Moderate
Canaud et al. (37)	31.5	NR	NR	100	Low
Kamman et al. (38)	36.6 ± 26.8	NR	11 (14.9)	100	Moderate
Piffaretti et al. (19)	24 ± 21	NR	2 (4.8)	100	Moderate
van der Weijde et al. (39)	42	NR	2 (2.0)	NR	Low
Xiang et al. (20)	39.9 ± 24.1	NR	1 (7.1)	100	Moderate
Delafontaine et al. (40)	NR	NR	NR	NR	Low
Bartos et al. (41)	11.1 ± 1.3	NR	NR	97	Low
Bianco et al. (42)	33.6	NR	7 (12.1)	100	Very low
D’Oria et al. (43)	NR	74 (10.3)	NR	NR	Moderate
Ramdon et al. (21)	NR	NR	NR	98.5	Moderate
Wang et al. (26)	12.7 ± 9.3	NR	NR	NR	Low
Johnson et al. (44)	25.4	NR	NR	100	Moderate
Parker et al. (45)	NR	2 (5.7)	NR	100	Very low
Xie et al. (46)	NR	NR	NR	NR	Low
Cumulative data	26.6 ± 2.4	9.4% 95%CI: 7.3–11.7%	6.9% 95%CI: 3.0–12.0%	99.4% 95%CI: 98.4–100.0%	

CI, Confidence interval; NR, not reported.

#### Late outcomes

The mean follow-up time was 12.9 months for a total of 322 patient-years in the ER cohort, while in the SR cohort, it was 26.6 months for a total of 1,717 patient-years of follow-up. The mean follow-up length in the three subgroups of the ER cohort were 22.8 months in chimney, 15.1 months in fenestration and 3.5 months in single-branched stent-grafts group, respectively. Only three of the SR studies reported a follow-up period exceeded 3 years. The cumulative rate of late reintervention after ER was 5.9% (95% CI: 1.9–11.4%) and 6.9% (95% CI: 3.0–12.0%) after SR. The restenosis rate during the follow-up period was 1.7% (95% CI: 0.0–9.9%; Table 1) in ER and 0.9% (95% CI: 0.0–4.9%) in SR. Pooled estimates of overall patency rates were 99.9% (95% CI: 98.1–100.0%) and 99.4% (95% CI: 98.4–100.0%), respectively (Table 2).

#### Subgroup analysis

The ER group was divided into three cohorts according to different methods of intervention, chimney grafts, fenestration group and single-branched stent-grafts. Subgroup analysis show that 30-day mortality was 1.6% (95% CI: 0.0–6.9%), 0.1% (95% CI: 0.0–2.1%) and 0.6% (95% CI: 0.0–4.1%), respectively. Stroke rate was 4.2% (95% CI: 0.0–16.8%) for chimney, 0.0% (95% CI: 0.0–1.1%) for fenestration and 0.0% (95% CI: 0.0–1.6%) for single-branched stent-grafts. No case of left limb ischemia has been reported in any subgroup. Pooled result indicated significant differences in the incidence of endoleaks among chimney, fenestration, and single-branched groups, with a cumulative data of 14.2% (95% CI: 0.0–44.6%), 10.2% (95% CI: 4.7–17.0%) and 3.9% (95% CI: 0.5–9.3%; Table 1). Notably, the chimney-grafts method showed a relatively high incidence of almost all the complications analyzed in this study, whereas single-branched stent-grafts showed relatively better outcomes. There were no significant subgroup differences for left arm claudication and cumulative patency rates among the ER group. Analysis of early reinterventions and restenosis rates were not feasible due to a lack of data.

### Data from comparative studies

#### Study selection

Six controlled retrospective studies were included, involving 438 patients contributed to comparative meta-analyses. All six included studies used bypass surgery as the method of LSA revascularization, with the chimney-grafts as the control group in three of the studies and the fenestration method as the control group in the remaining three studies.

#### Outcomes

No statistically significant results were observed among the six studies described above, including 30-day mortality, stroke, endoleaks and spinal cord ischemia, etc. (Supplementary Figures 1–8).

## Publication bias

In terms of funnel plot and Egger testing, the results of the visual analysis did not reveal any indications of publication bias within the SR group. Only left arm claudication was identified as a significant risk of bias in the ER studies (Supplementary Figures 9–38).

## Discussion

Over the last decade, several studies have suggested the potential benefits of LSA revascularization, a procedure that has been shown to reduce the incidence of postoperative complications compared to direct coverage (47–49). Since there is still no consensus on the advantages and disadvantages of different methods of LSA revascularization, this systematic review was conducted to attempt to answer this question by comparing the complications associated with each technique.

Our study reviewed contemporary methods and clinical outcomes of 2,759 patients from 28 individual studies for LSA revascularization during TEVAR, indicating that performing ER or SR during TEVAR has comparable effects on the outcomes. LSA revascularization with open surgical procedures used to be the most widely applied revascularization approach (50).

Although LSA revascularization through bypass or transposition surgery is technically sophisticated, it entails excessive trauma and compromises the minimally invasive concept of TEVAR. Besides, open surgery can lead to potential local complications, such as irritation of the brachial plexus nerve (41). In comparison, LSA revascularization using endovascular approaches is in line with the minimally invasive concept of the TEVAR procedure and the technology is well established (29). Actually, the indications for complete endovascular aortic reconstruction are also expanding (51). Therefore, we cautiously believe that the ER methods may have some advantages in avoiding unnecessary incisions and trauma to patients.

Another advantage of ER is the shorter surgical time requirement as compared to open surgery (46). When performing ER, the stent-graft is also more tolerant of DSA imaging accuracy and proficiency of manipulation (52).

In terms of ER of LSA, currently there are three main methods: chimney techniques, *in situ* fenestration procedures, and single-branched stent grafts. The subgroup analysis indicates that all three methods present acceptable clinical results, although there are still some notable differences in some aspects. This subtle difference merits a careful examination in order to identify their strengths and latent weaknesses.

Our main focus was on stroke and endoleak. Specifically, when the chimney method is applied to LSA revascularization, it results in an increased rate of postoperative complications. We identified that the incidence of stroke (4.2%; 95% CI: 0.0–16.8%) and endoleak (14.2%; 95% CI: 0.0–44.6%) was markedly higher in the chimney group. In comparison, LSA revascularization with single-branched stent-grafts demonstrated a considerably lower perioperative complication rates than expected, with no perioperative strokes recorded and the incidence of endoleak was only 3.9% (95% CI: 0.5–9.3%). In particular, it should be noted that despite this result appearing to be extremely favorable, the findings obtained by this study have a great deal of bias, as the number of single-branch stents reported is the lowest of all techniques.

Fenestrated technique has been associated with modest outcomes, with the incidence of stroke was 0.0% (95% CI: 0.0–1.5%) and the incidence of endoleak was 10.2% (95% CI: 4.7–17.0%), which was relatively high compared to the result of the single-branched stent-graft group.

The chimney technique is a widely accepted and utilized method for LSA revascularization in TEVAR. It is a much more technically accessible approach to implement than fenestration technique and using single-branched stent-graft, yet a mounting number of studies suggest that it may increase the risk of endoleaks and reintervention rates (53, 54). According to one relevant publication, endoleaks are responsible for the majority of secondary interventions after TEVAR (55). In the current review, the perioperative endoleak rate of the chimney technique is 14.2% (95% CI: 0.0–44.6%), significantly higher than the average incidence in the ER (8.3%; 95% CI: 2.1–17.1%) and SR (11.2%; 95% CI: 4.4–20.5%) groups. It is also the highest of the three ER methods. Even though early reintervention rates were not recorded in the included studies, one previous study reported a rate of 11% of type I endoleaks associated with the chimney technique, and 42% of these patients required early reintervention (56). Moreover, we also found a late reintervention rate of 8.6% (95% CI: 2.0–18.2%) among the chimney group, which is higher than the cumulative rate of ER (5.9%; 95% CI: 1.9–11.4%) and SR (6.9%; 95% CI: 3.0–12.0%) group. Based on the findings of this analysis, it has been speculated that the high incidence of endoleaks may be one of the main factors leading to reintervention. Considering that reintervention rates were also higher in groups with a higher incidence of endoleak, we felt that it was essential to consider the potential risk associated with the chimney method.

Fenestration is another technique commonly used for LSA revascularization. There are two types of fenestration techniques, pre-fenestration and *in situ* fenestration, each of which may be appropriate in certain clinical situations. In the current meta-analysis, all the cases in other studies except two were totally complemented *in situ* fenestration technique (26, 46). An increasing number of studies have described the high repeatability and low perioperative mortality and morbidity of this procedure (25, 57, 58). Current evidence obtained from this analysis suggests that the incidence of 30-day mortality and early outcomes in the fenestrated cohort is close to the overall results of ER group. It is worth mentioning that the incidence of endoleak of the fenestration method is 10.2% (95% CI: 4.7–17.0%), which is relatively close to the average incidence of endoleak in the ER group derived in this analysis, while the incidence level is lower compared to the chimney group. This variation is understandable because the fenestration technique does not cause gutter endoleaks and has a relatively sufficient landing zone, which explains the significantly lower incidence of endoleaks reported in previous studies (59, 60). In comparison, it should be noted that the fenestration technique may result in an increased incidence of type III endoleak, which may be explained by the fact that the sealing zone between the connecting stent-graft and the LSA is relatively short and requires strong adhesion to the aortic wall (59). In general, considering the relatively low fenestration-related morbidity demonstrated shown in this analysis, the fenestration technique remains a safe, reproducible and durable procedure for LSA revascularization.

The single-branched stent-graft includes a main body and a branch graft that by design reduces the risk of endoleaks (28). It has been hypothesized that due to the superior stability of the single-branch stent and its ability to maintain structural integrity, the incidence of endoleaks should be reduced. This is in accordance with the results obtained in this analysis. According to results from a previous multicenter study, single-branched stent-grafts were found to be an effective technique for reconstructing the distal aortic arch (61). Additionally, we also noticed that almost all perioperative complications showed promising results with single-branched stent during TEVAR, including no occurrence of stroke, spinal cord ischemia, or left arm claudication during the in-hospital period or 30 days after the procedure. Moreover, Fang et al. noted that single-branched stent-grafts are simpler to implement than fenestration, suggesting that the method has good feasibility (29). Limited evidence so far suggests that the method has very promising potential for broader application. Nevertheless, this does not necessarily mean that the single-branch stent method is the most effective option. From our current review, the postoperative follow-up time of the cohort with branched stent-grafts was considerably shorter than the other groups undergoing LSA revascularization. As a result, the lack of long-term data is likely to have contributed to the underlying bias. Additionally, custom stent grafts are often required to compensate for the heterogeneity of the aortic arch anatomy, complicating the establishment of large-scale clinical studies.

Due to the distinct and independent characteristics of each method, we cannot extrapolate which is the more reliable solution. Their full potential has yet to be demonstrated, and it is reasonable to expect more details to be documented and worked out in clinical practice. Taking all these factors into account, a feasible option for the method of LSA revascularization during TEVAR should be carefully chosen based on the patients’ indications, while considering efficacy, practicality, and the practitioners’ expertise. As a result, this is probably the best way to ensure a viable landing zone while maintaining the blood supply to the LSA, allowing patients to benefit from TEVAR as fully as possible.

## Limitations

Several limitations were identified in the current analysis that need to be carefully considered. Firstly, since no relevant randomized controlled trials (RCTs) were retrieved, all of the included studies were retrospective in design. Secondly, the data collected are primarily based on English-language studies, and therefore information from published research papers in other languages is not included. Thirdly, the heterogeneity between groups and subgroups is relatively significant, and the amount of literature used to conduct subgroup analyses varies considerably. Finally, the baseline characteristics of the patients that included in the analysis were not fully taken into account, furthermore, the data used in the analysis are partially missing and incomplete, contributing to bias in this study. In light of the above limitations, additional research is needed in the future to derive more accurate and reliable results.

## Conclusion

As new endovascular devices and techniques are developed, complete ER is expected to become the standard of care for the treatment of LSA in the near future. It will, however, take a considerable amount of time for these alternatives to be verified. We cannot yet be certain whether ER will be able to completely replace SR, as there are still a number of uncertainties to be resolved. This review emphasizes the critical role of RCT research in providing insight into the manner in which LSA revascularization is performed during TEVAR. Further well-designed and large-scale studies are required to give more persuasive evidence regarding the optimal technique to LSA revascularization and to serve as the foundation for new treatment guidelines.

## Data availability statement

The original contributions presented in this study are included in the article/Supplementary material, further inquiries can be directed to the corresponding authors.

## Author contributions

YucZ designed the study, collected, analyzed, and wrote the first draft of the manuscript. XX and YY collected data from the included studies and evaluated them according to the aforementioned criteria. CH, EW, and YufZ evaluated and proofread the data obtained from the studies included in this review. PL, ZL, and FM reviewed the statistical analysis methods and results that were conducted. WF and LW conceived and designed the study, coordinated and monitored its conduct, and carefully revised the final manuscript. All authors read and approved the final manuscript.

## Footnotes

1
https://www.r-project.org/
