# Supplementary material for: Comparison of techniques for left subclavian artery preservation during thoracic endovascular aortic repair: A systematic review and single-arm meta-analysis of both endovascular and surgical revascularization
Source: Front Cardiovasc Med. 2022 Sep 15;9:991937. doi: 10.3389/fcvm.2022.991937 (PMC9520576; doi:10.3389/fcvm.2022.991937)
Supplement: Supplementary file 1 [file Data_Sheet_1.docx]

Supplementary Material

# Supplementary Figures


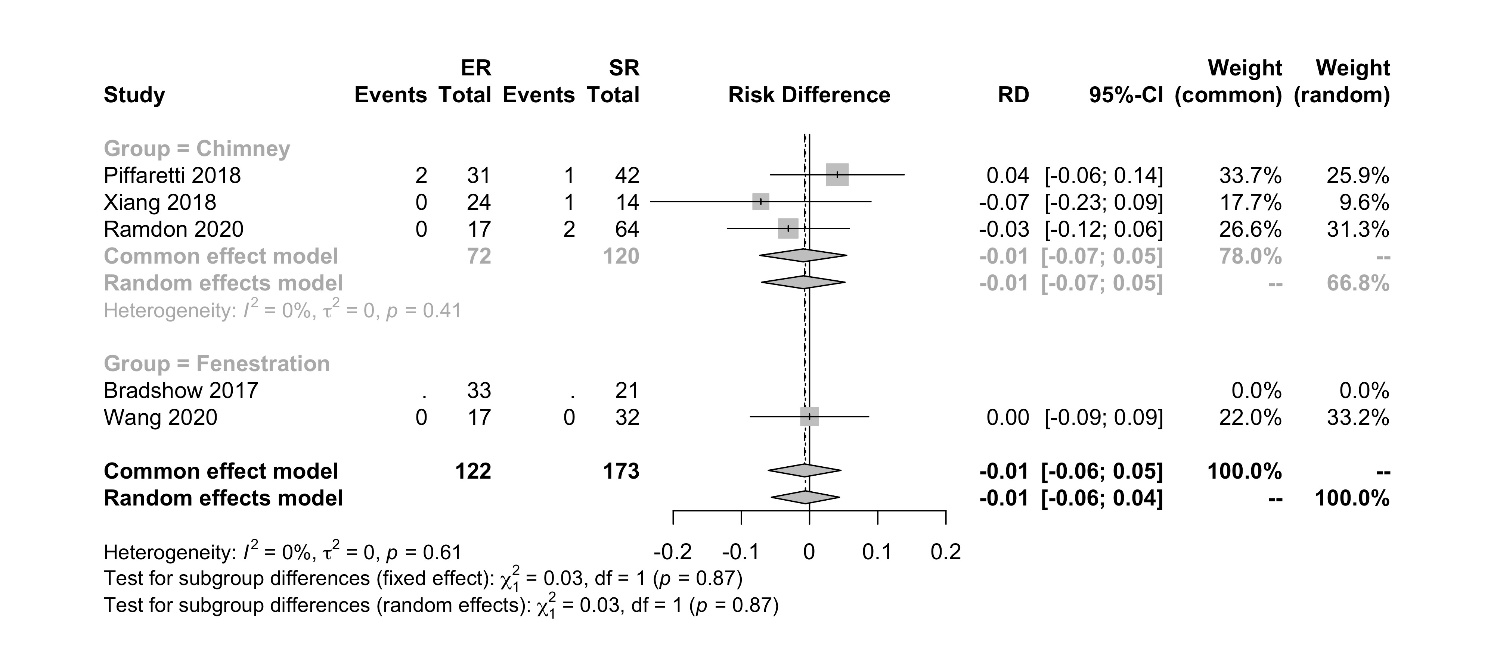


**Supplementary Figure 1.** 30-mortality.


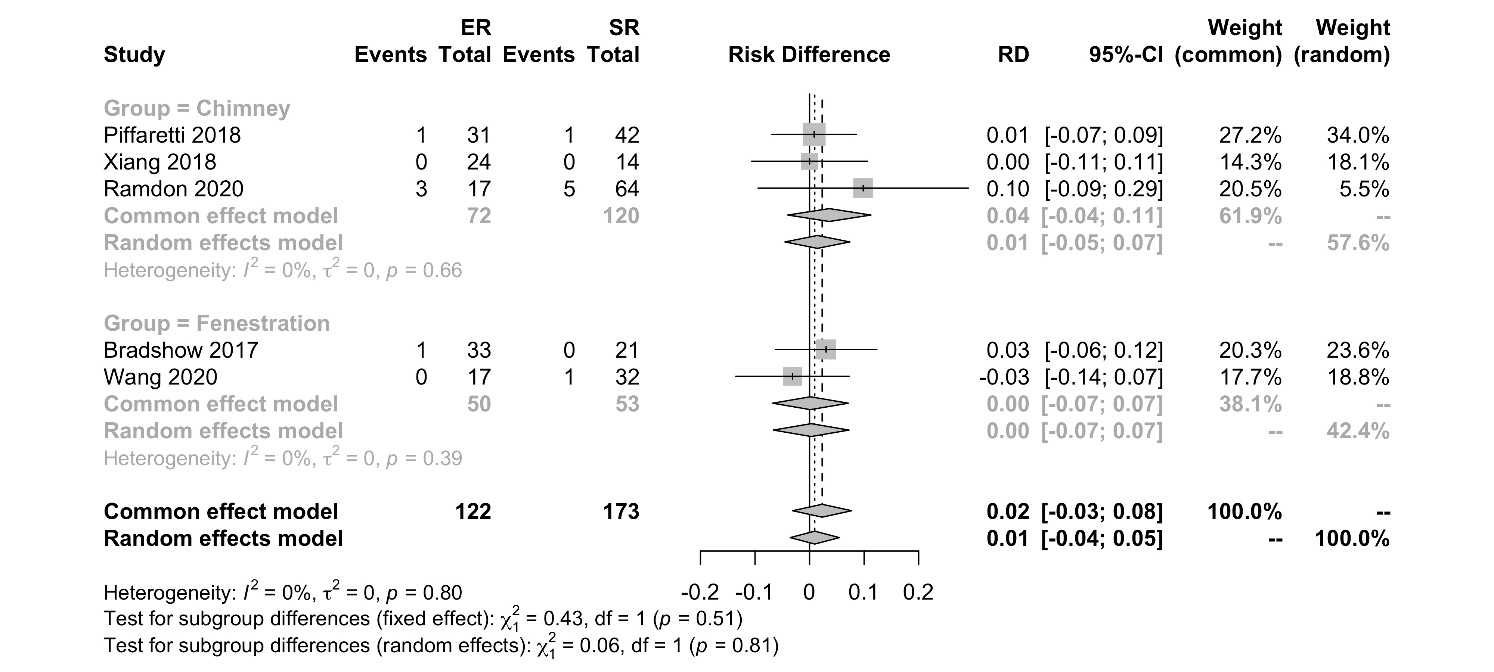


**Supplementary Figure 2.** Stroke.


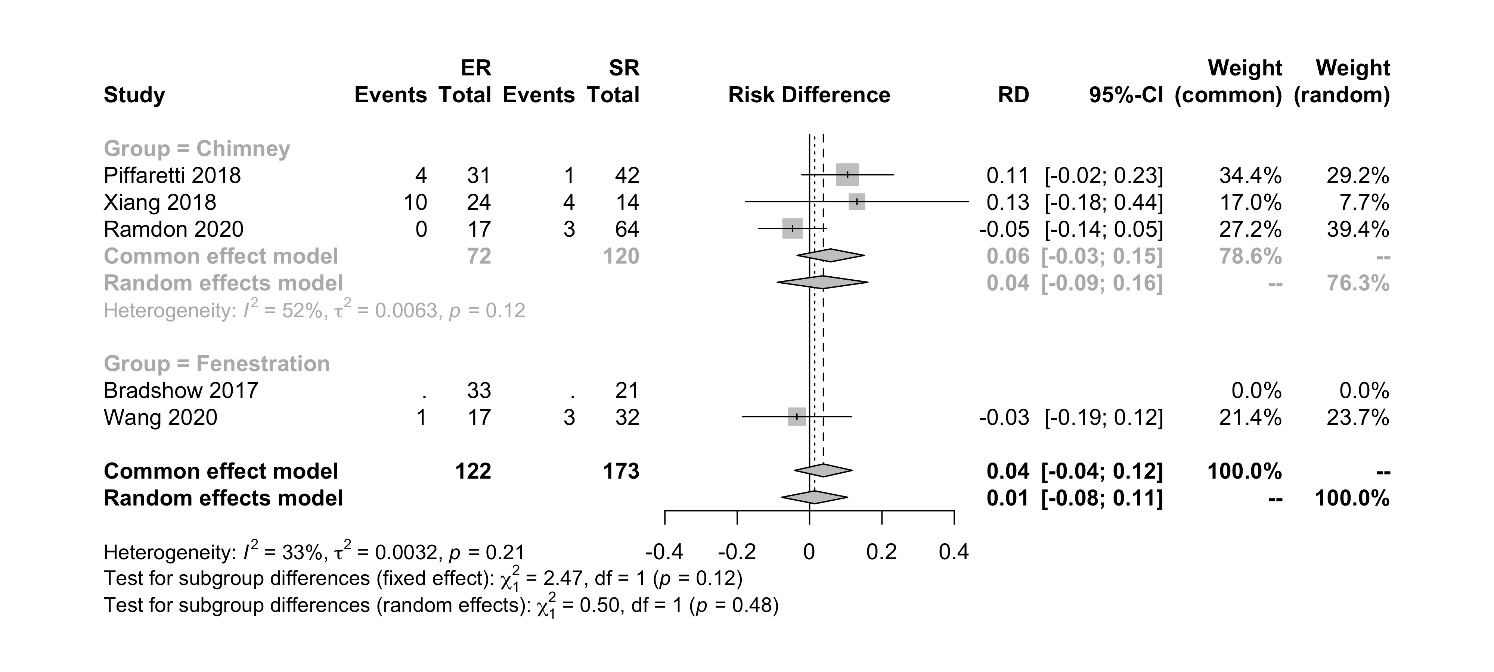


**Supplementary Figure 3.** Endoleak.


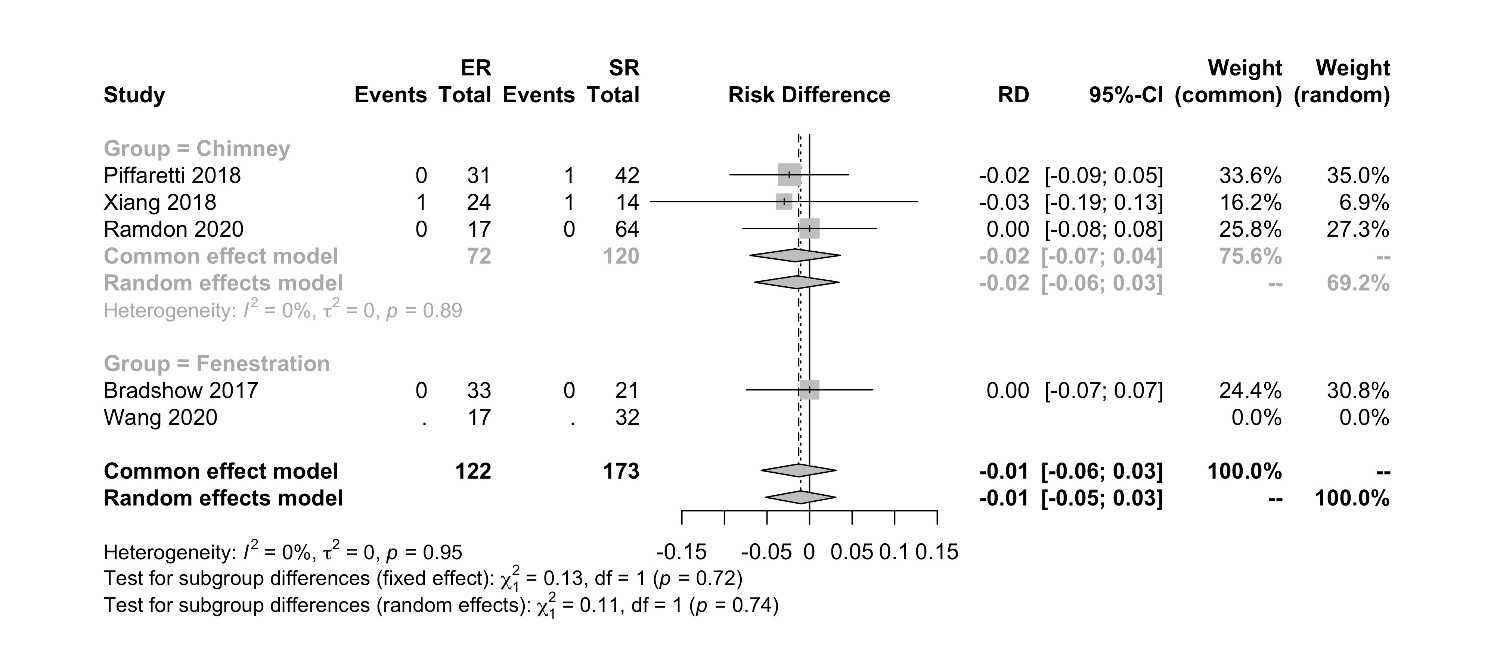


**Supplementary Figure 4.** Spinal cord ischemia.


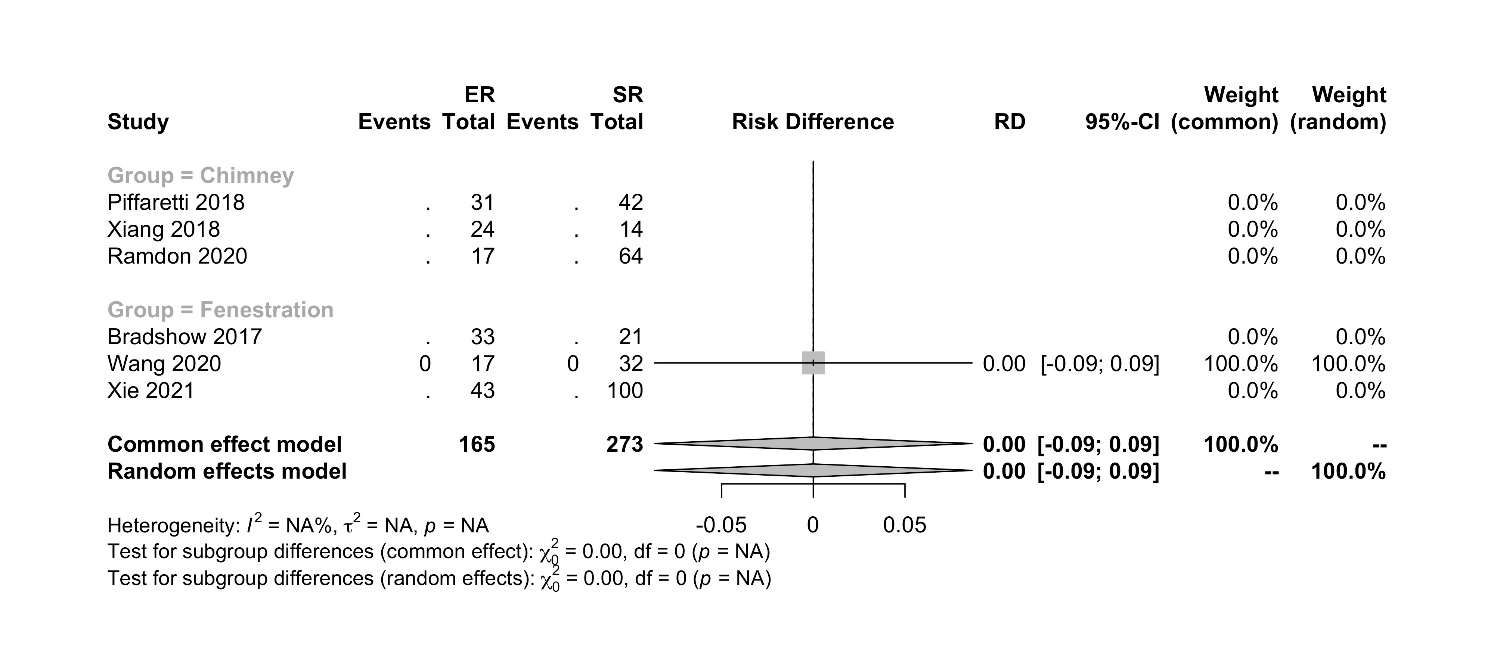


**Supplementary Figure 5.** Restenosis.


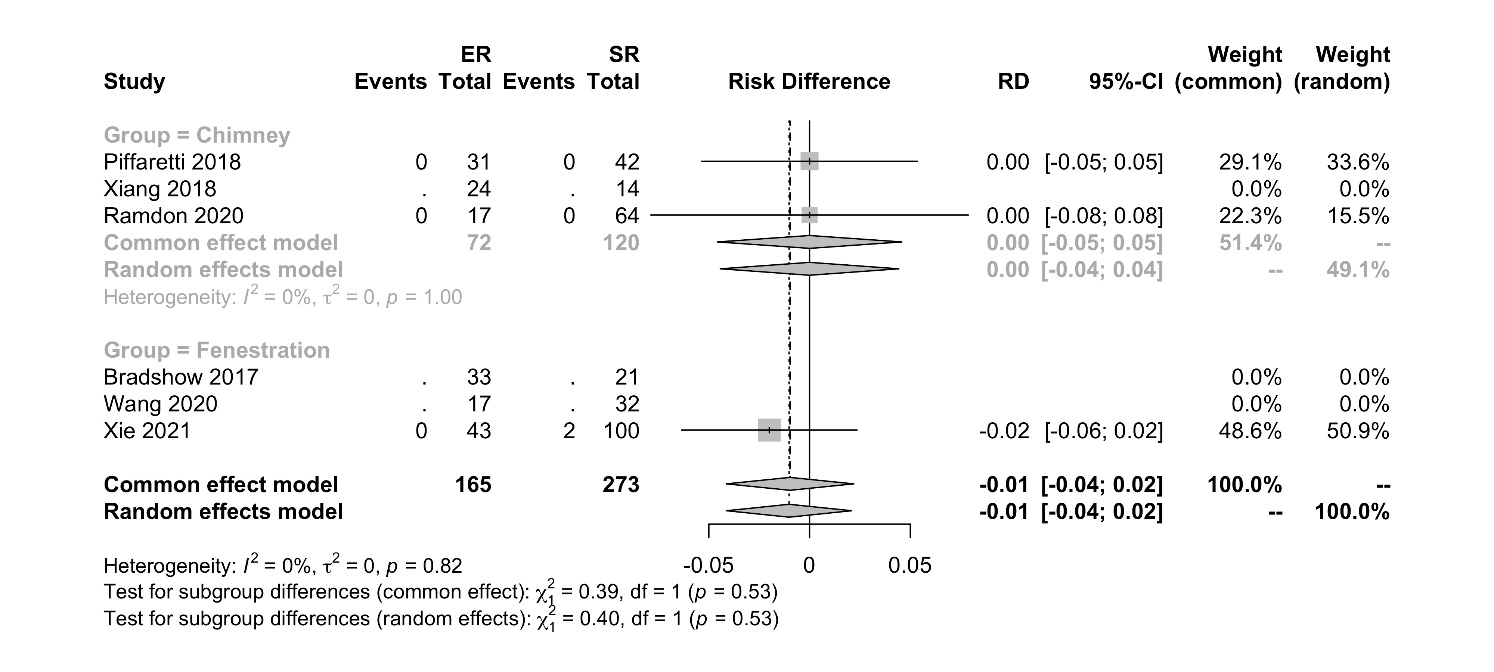


**Supplementary Figure 6.** Left arm claudication.


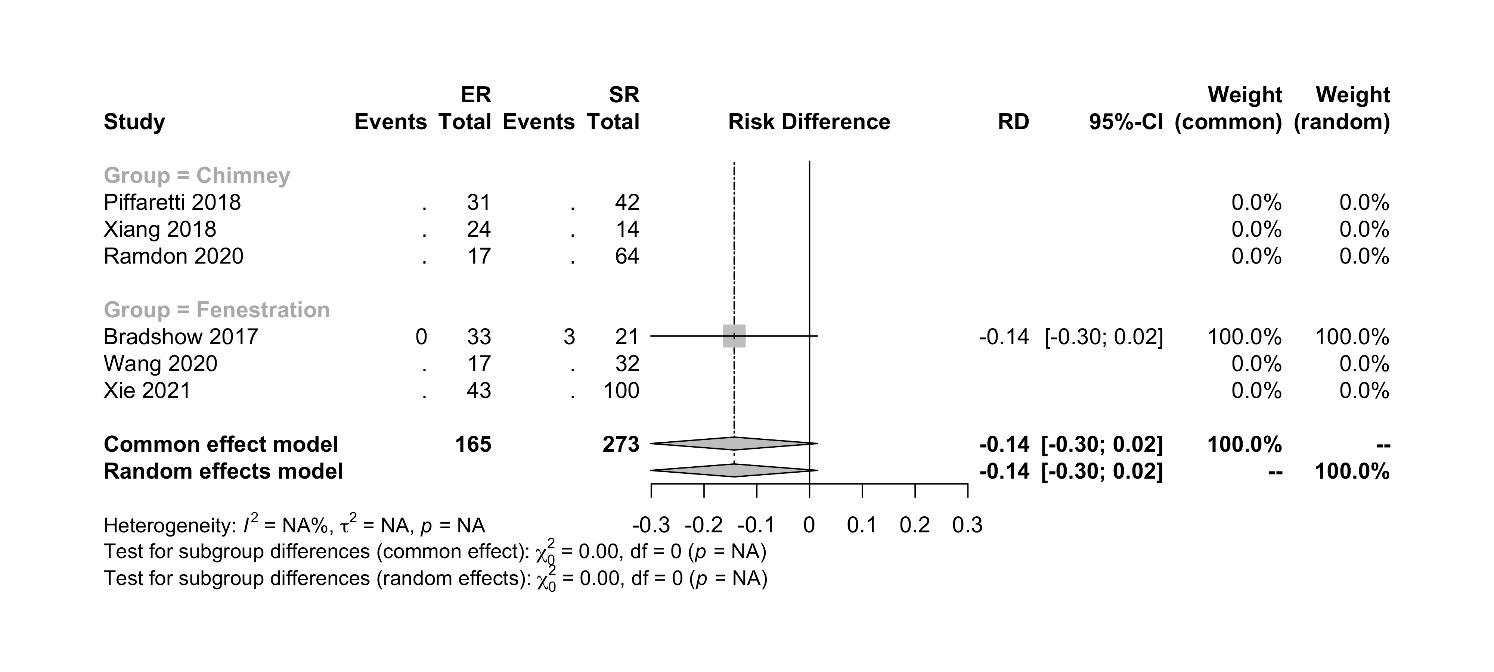


**Supplementary Figure 7.** Early reintervention.


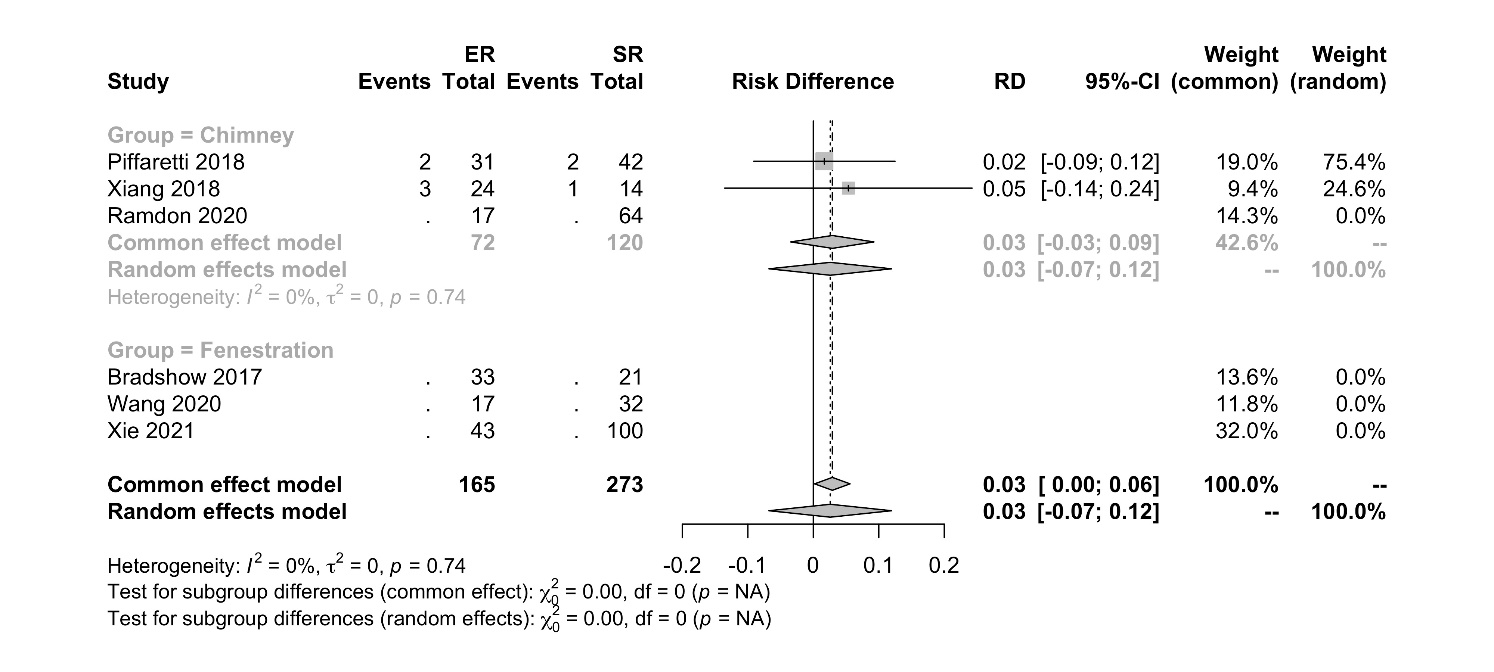
**Supplementary Figure 8.** Late reintervention.

**Funnel plots and results of the egger test. (Endovascular revascularization)**


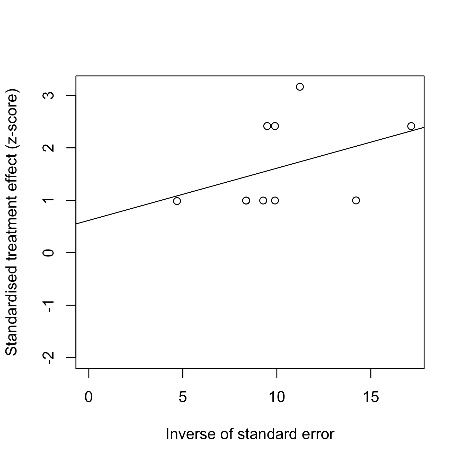

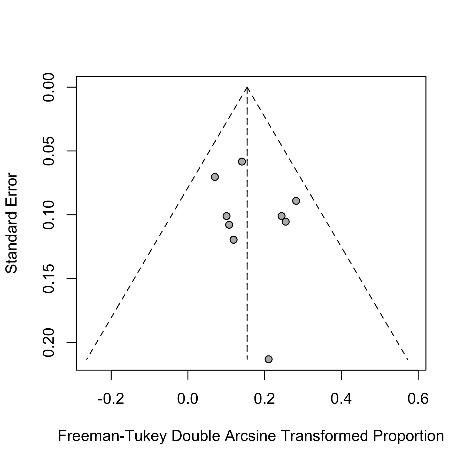


**Supplementary Figure 9.** Egger test of 30-day mortality. (P value = 0.5032)

**Supplementary Figure 10.** Funnel plot of 30-day mortality.


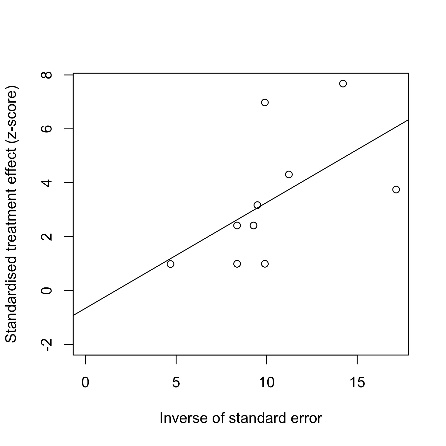

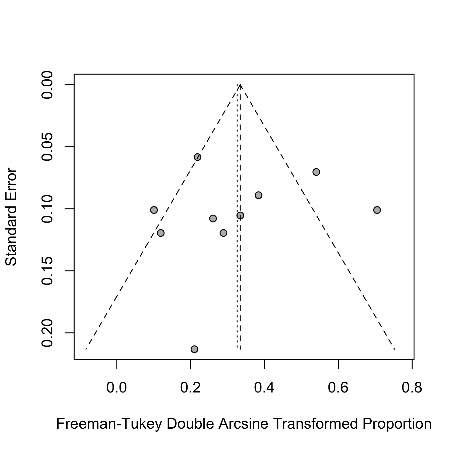


**Supplementary Figure 11.** Egger test of endoleak. (P value = 0.7764)

**Supplementary Figure 12.** Funnel plot of endoleak.


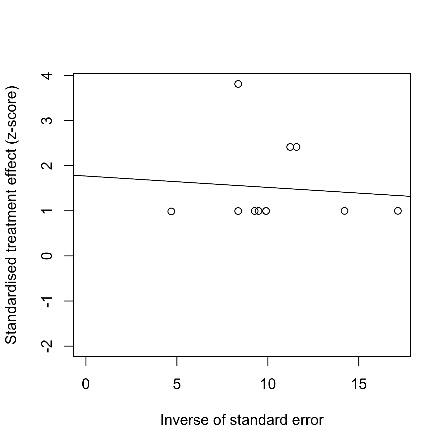

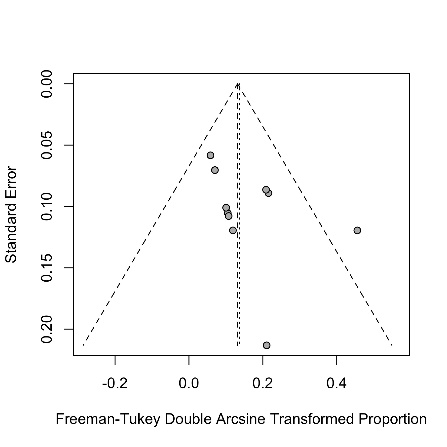


**Supplementary Figure 13.** Egger test of stroke. (P value = 0.1268)

**Supplementary Figure 14.** Funnel plot of stroke.


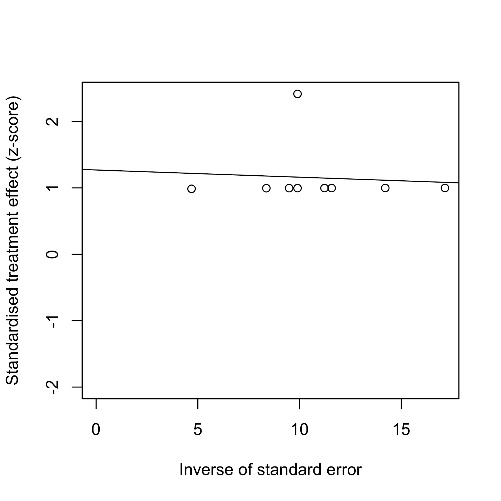

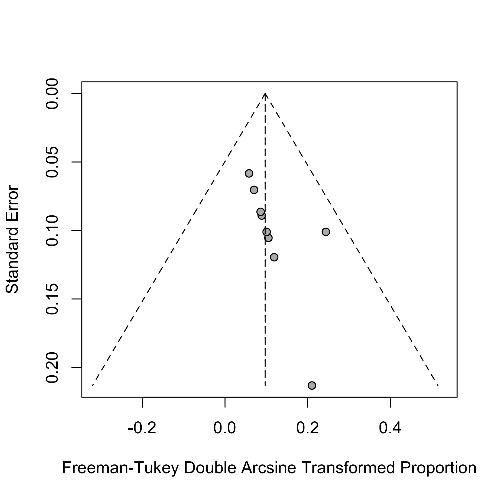


**Supplementary Figure 15.** Egger test of spinal cord ischemia. (P value = 0.0605)

**Supplementary Figure 16.** Funnel plot of spinal cord ischemia.


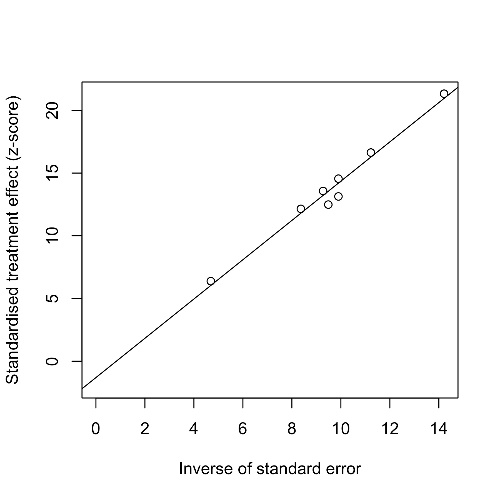

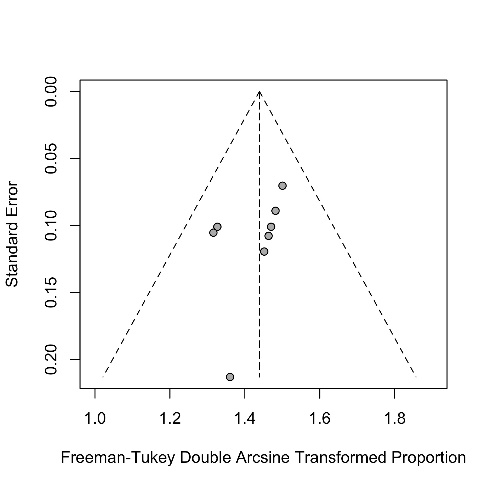


**Supplementary Figure 17.** Egger test of restenosis. (P value = 0.2404)

**Supplementary Figure 18.** Funnel plot of restenosis.


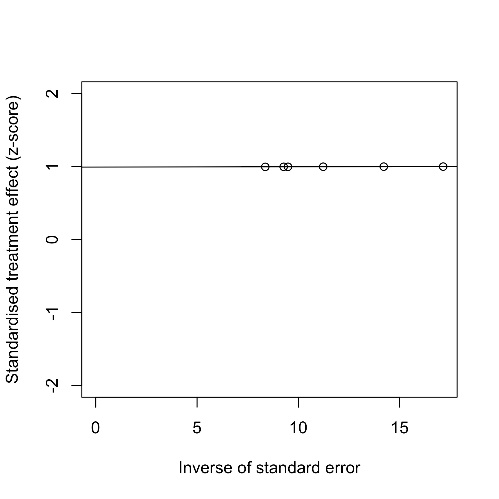

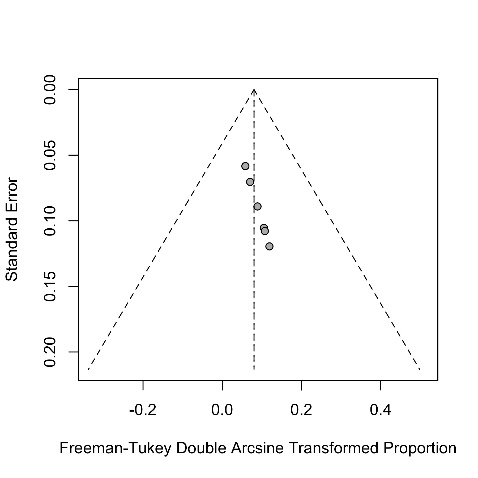


**Supplementary Figure 19.** Egger test of left arm claudication. (P value = 0.0001)

**Supplementary Figure 20.** Funnel plot of left arm claudication.


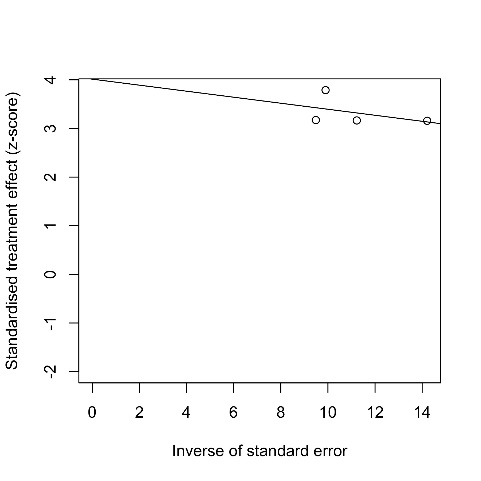

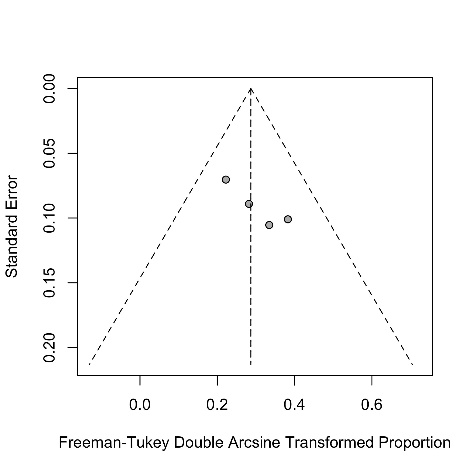


**Supplementary Figure 21.** Egger test of late reintervention. (P value = 0.0631)

**Supplementary Figure 22.** Funnel plot of late reintervention.

**Funnel plots and results of the egger test. (Open revascularization)**


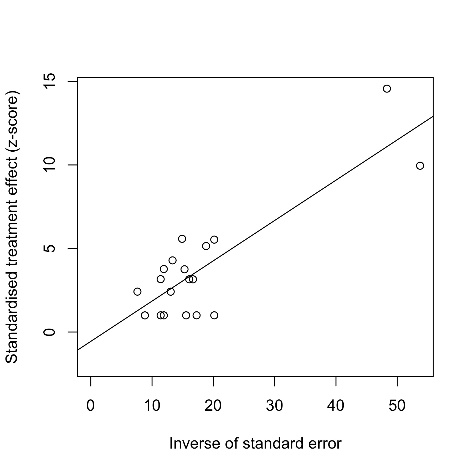

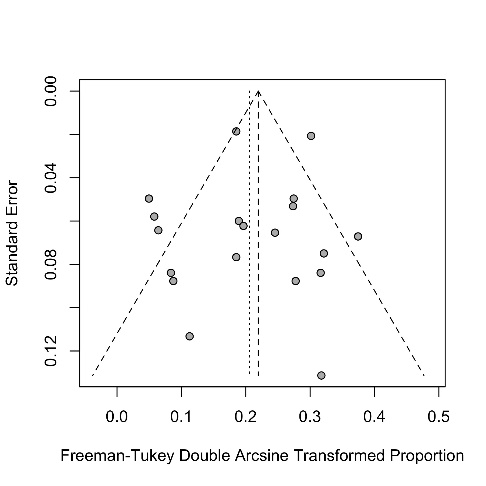


**Supplementary Figure 23.** Egger test of 30-day mortality. (P value = 0.4922)

**Supplementary Figure 24.** Funnel plot of 30-day mortality.


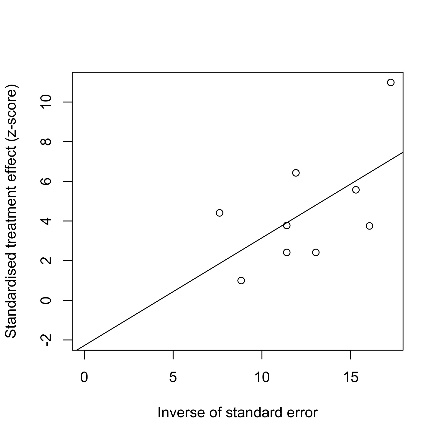

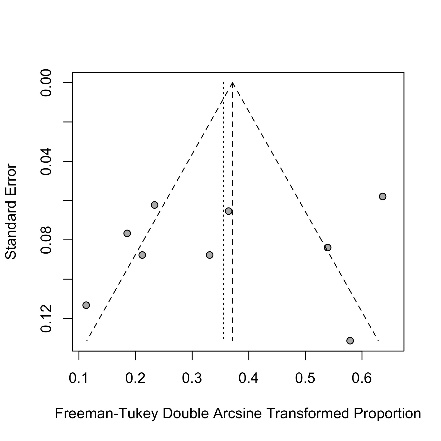


**Supplementary Figure 25.** Egger test of endoleak. (P value = 0.5417)

**Supplementary Figure 26.** Funnel plot of endoleak.


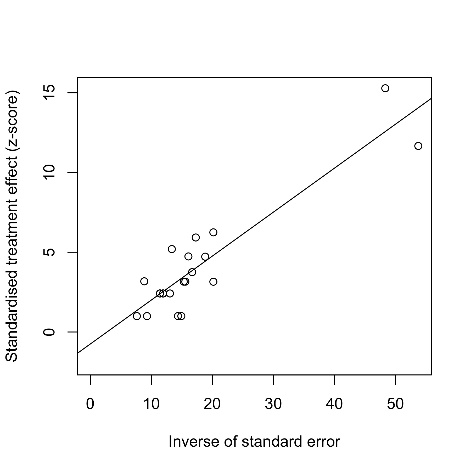

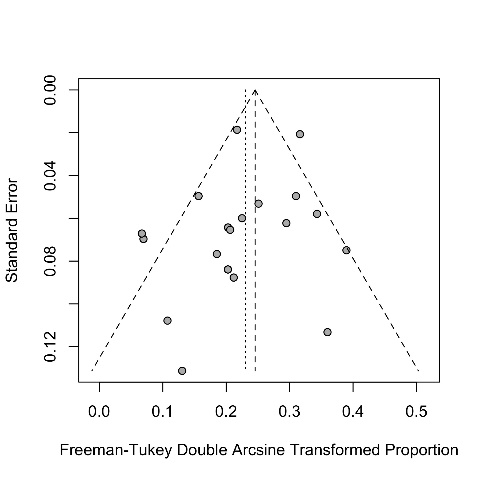


**Supplementary Figure 27.** Egger test of stroke. (P value = 0.2238)

**Supplementary Figure 28.** Funnel plot of stroke.


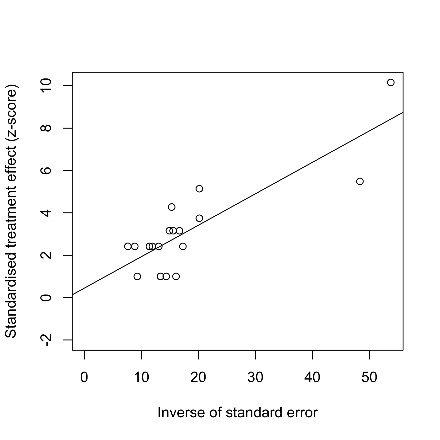

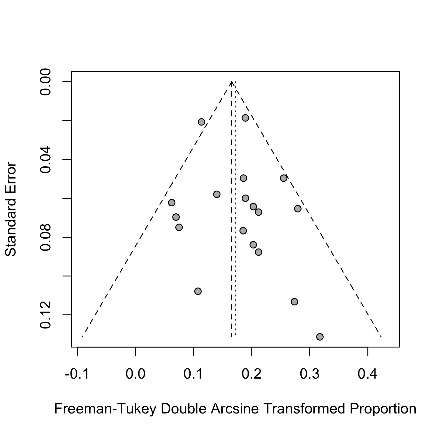


**Supplementary Figure 29.** Egger test of spinal cord ischemia. (P value = 0.3995)

**Supplementary Figure 30.** Funnel plot of spinal cord ischemia.


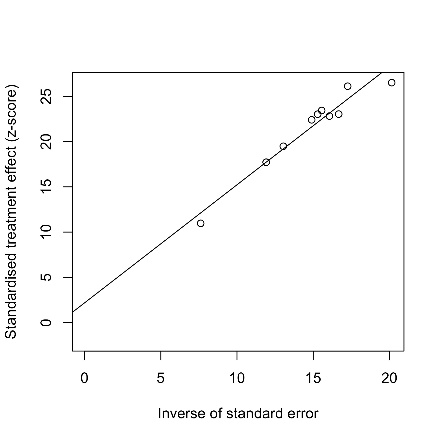

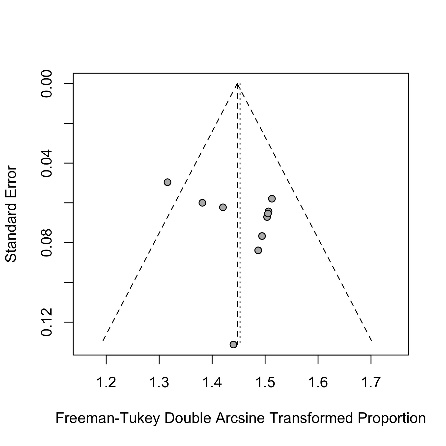


**Supplementary Figure 31.** Egger test of restenosis. (P value = 0.1855)

**Supplementary Figure 32.** Funnel plot of restenosis.


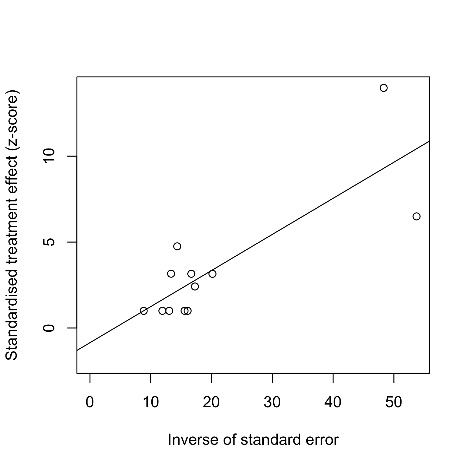

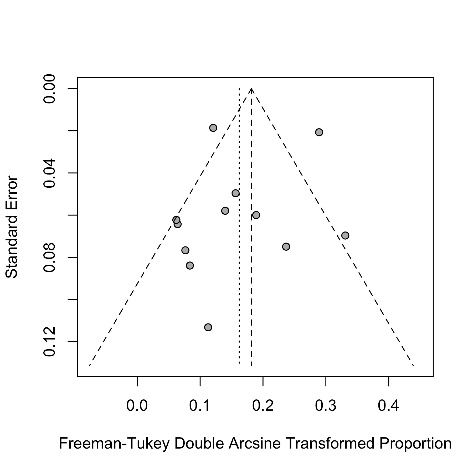


**Supplementary Figure 33.** Egger test of left arm claudication. (P value = 0.4855)

**Supplementary Figure 34.** Funnel plot of left arm claudication.


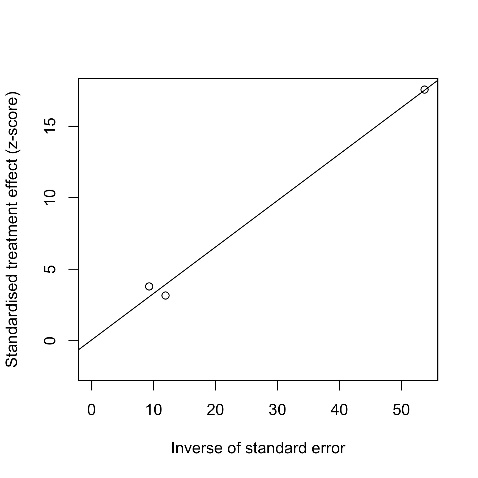

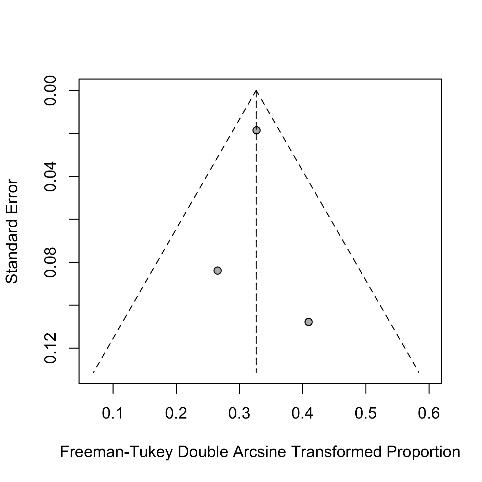


**Supplementary Figure 35.** Egger test of early reintervention. (P value = 0.9618)

**Supplementary Figure 36.** Funnel plot of early reintervention.


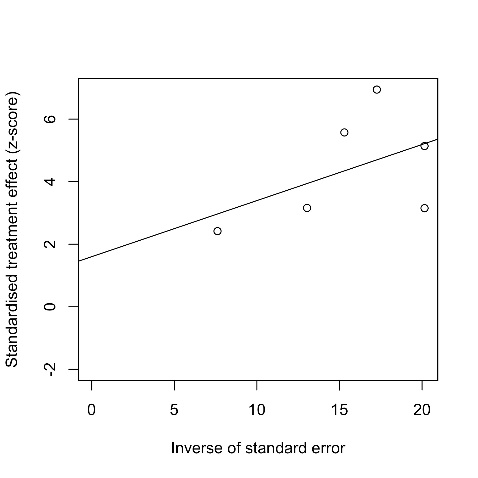

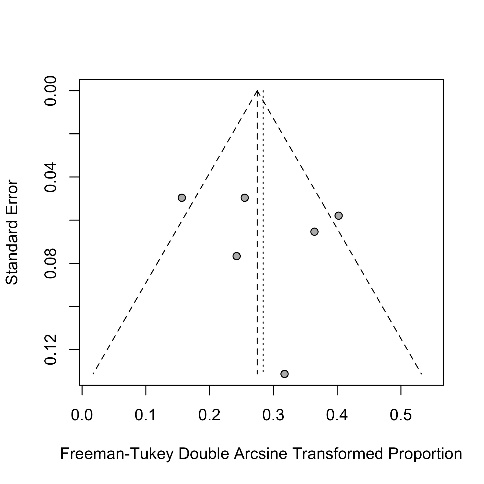


**Supplementary Figure 37.** Egger test of late reintervention. (P value = 0.5700)

**Supplementary Figure 38.** Funnel plot of late reintervention.
